# Retraction: Self-association of L-periaxin occurs *via* its acidic domain and NLS2/NLS3, and affects its trafficking in RSC96 cells

**DOI:** 10.1039/d1ra90105b

**Published:** 2021-04-23

**Authors:** Yenan Yang, Min Liang, Yawei Shi

**Affiliations:** Institute of Biotechnology, Key Laboratory of Chemical Biology, Molecular Engineering of Ministry of Education, Shanxi University Taiyuan 030006 P. R. China yangyenan@foxmail.com 1551193739@qq.com yaweishi@sxu.edu.cn +86-351-7018268

## Abstract

Retraction of ‘Self-association of L-periaxin occurs *via* its acidic domain and NLS2/NLS3, and affects its trafficking in RSC96 cells’ by Yenan Yang *et al.*, *RSC Adv.*, 2017, **7**, 44112–44123, DOI: 10.1039/C7RA06853K

The Royal Society of Chemistry, with the agreement of the authors, hereby wholly retracts this *RSC Advances* article due to concerns with the reliability of the data in the published article. The authors confirmed that the western blot images in Fig. 2, 3, 4, 6 and 7 had been inappropriately manipulated. Given the significance of the concerns about the validity of the data, the findings presented in this paper are no longer reliable.

Signed: Yenan Yang, Min Liang and Yawei Shi

Date: 8^th^ April 2021

Retraction endorsed by Laura Fisher, Executive Editor, *RSC Advances*

## Supplementary Material

